# Announcing the New Website of Balkan Medical Journal

**DOI:** 10.4274/balkanmedj.2017.5.0002

**Published:** 2017-09-29

**Authors:** Zafer Koçak, Çetin Hakan Karadağ

**Affiliations:** 1 Department of Radiation Oncology, Trakya University School of Medicine, Edirne, Turkey; 2 Department of Pharmacology, Trakya University School of Medicine, Edirne, Turkey

At the end of the last year, we, editorial team, decided to redesign our website to better serve our readers, reviewers and authors. After much hard work, we are excited to officially announce the new and improved Balkan Medical Journal website. You can now reach us at http://balkanmedicaljournal.org/

We thought that it was important to renew the website to reach a wider audience. So, we wanted to have good, clear, easy-to-follow navigation throughout our website. The new website will remain both open access and free of article processing charges. We hope that the website will respond better to our readers’ needs and interests.

Key features of the new website include:

• Drop down menus to make easier to navigate and improve content layout and design,

• Pre-submission inquiry to allow author to have quick feedback from Balkan Medical Journal’s Editors regarding the suitability of a manuscript for the journal,

• Image corner which is created from clinical images published in the latest issues for teaching and training purposes,

• News section to stay up-to-date on the latest with health news and health science,

• Articles section which is composed of selected publications from current issue,

• Email alert feature to keep you up-to-date with content of the journal,

• We provide you with necessary information about citing in How to Cite,

• Advanced Search gives you opportunity to find what you seek more easily,

• Select articles you want and make the site to show them with View Selected Articles,

• You can share your favorite articles with your colleagues on social media with Share feature.

We would like to thank our publisher, Galenos Publishing House, for their tremendous support and effort.

For any questions, suggestions, feedback or comments, please contact us via E-mail.

## Figures and Tables

**Figure 1:**
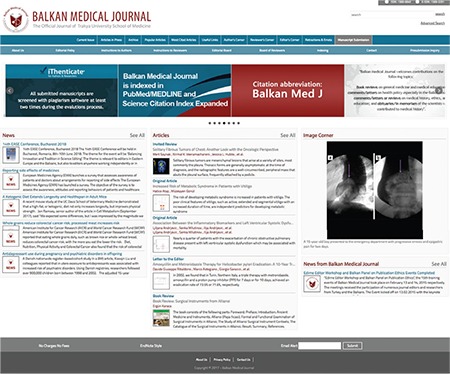
http://balkanmedicaljournal.org/

